# Motion processing across multiple topographic maps in the electrosensory system

**DOI:** 10.1002/phy2.253

**Published:** 2014-03-20

**Authors:** Navid Khosravi‐Hashemi, Maurice J. Chacron

**Affiliations:** ^1^ Department of Physiology McGill University Montreal Quebec Canada; ^2^ Department of Physics McGill University Montreal Quebec Canada

**Keywords:** Neural coding, pyramidal neuron, velocity tuning, weakly electric fish

## Abstract

Animals can efficiently process sensory stimuli whose attributes vary over orders of magnitude by devoting specific neural pathways to process specific features in parallel. Weakly electric fish offer an attractive model system as electrosensory pyramidal neurons responding to amplitude modulations of their self‐generated electric field are organized into three parallel maps of the body surface. While previous studies have shown that these fish use parallel pathways to process stationary stimuli, whether a similar strategy is used to process motion stimuli remains unknown to this day. We recorded from electrosensory pyramidal neurons in the weakly electric fish *Apteronotus leptorhynchus* across parallel maps of the body surface (centromedial, centrolateral, and lateral) in response to objects moving at velocities spanning the natural range. Contrary to previous observations made with stationary stimuli, we found that all cells responded in a similar fashion to moving objects. Indeed, all cells showed a stronger directionally nonselective response when the object moved at a larger velocity. In order to explain these results, we built a mathematical model incorporating the known antagonistic center–surround receptive field organization of these neurons. We found that this simple model could quantitatively account for our experimentally observed differences seen across E and I‐type cells across all three maps. Our results thus provide strong evidence against the hypothesis that weakly electric fish use parallel neural pathways to process motion stimuli and we discuss their implications for sensory processing in general.

## Introduction

Animals must efficiently process incoming sensory stimuli with widely varying intensities using sensory neurons with very limited output ranges in order to successfully interact with their environment. Parallel processing of sensory information by distinct neural circuits appears to be a common strategy used across modalities including auditory (Takahashi et al. 1984; Oertel 1999; Gelfand 2004; MacLeod and Carr 2007), visual (Marr 1982; Livingstone and Hubel 1987; Merigan and Maunsell 1993; Wassle 2004), and electrosensory (Carr and Maler 1986; Bell and Maler 2005; Kawasaki 2005; McGillivray et al. 2012) to code for different stimulus attributes.

**Figure 1:**
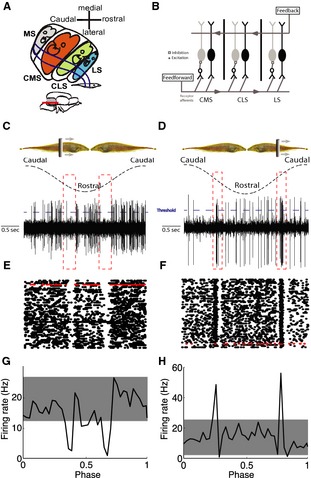
ELL pyramidal neurons respond to moving objects. (A) Sketch of a horizontal section through the right ELL showing the oblique orientation of the maps and the topographic relationship with the skin surface. Inset: lateral view of the brain with the ELL shown in gray. The red line indicates the dorsoventral level of the horizontal section. The medial segment (MS) receives input from ampullary electroreceptors, whereas the other three segments receive input from tuberous electroreceptors (CMS, centromedial segment; CLS, centrolateral segment; LS, lateral segment). The “pisciculi” of MS and CMS and of CLS and LS abut ventrally, the maps of CMS and CLS abut dorsally (after Carr et al. 1982). (B) Schematic of the ELL anatomy showing feedforward and feedback inputs onto ELL pyramidal neurons. Each peripheral electroreceptor afferent trifurcates to contact pyramidal cells within CMS, CLS, and LS. Within each segment, E cells receive direct excitation from these afferents, whereas I cells receive di‐synaptic inhibition via granule cell (GC) interneurons. ELL pyramidal cells also receive large amounts of feedback from higher brain centers. (C and D) Schematic showing the moving object stimulus (top) with one full cycle of movement starts at the caudal end, moves to rostral, and back to caudal, for example, I‐ and E‐type pyramidal cells, respectively. The threshold used to detect spikes is also shown (dashed blue lines). (E and F) Raster plots showing the responses from these two same cells to repeated cycles of movement with the trials corresponding to panels C,D in red. (G and H) Peristimulus time histograms (PSTH) from these same two cells.

Gymnotiform wave‐type weakly electric fish generate quasi‐sinusoidal electric fields through the electric organ discharge and monitor perturbations through electroreceptors scattered on their skin (Chacron et al. 2011). These perturbations cast electric images onto the skin where tuberous electroreceptor organs respond to changes in EOD amplitude and contact pyramidal neurons in the electrosensory lateral line lobe (ELL) (Scheich et al. 1973). In particular, each afferent axon trifurcates and makes synaptic contact with pyramidal neurons located within three parallel somatotopic maps of the body surface: the centromedial segment (CMS), centrolateral segment (CLS), and lateral segment (LS) (Fig. 1A) (Heiligenberg and Dye 1982). There are two types of ELL pyramidal neurons within each map: E cells respond with increasing firing rate while I cells respond with decreasing firing rate to increases in EOD amplitude and are analogous to the ON‐ and OFF‐type cells found in other systems, respectively (Maler et al. 1981; Saunders and Bastian 1984) (Fig. 1B). Pyramidal neurons receive large amounts of feedback that contribute in part to their receptive field structure (Chacron et al. 2003, 2005b) and to shape pyramidal cell responses to sensory input (Bastian 1986; Bastian et al. 2004; Chacron et al. 2005b; Bol et al. 2011). While there are clear differences between the receptive field structures of ELL pyramidal cells across segments (Shumway 1989a), the observed differences in frequency tuning appear to be mostly caused by differences in membrane conductances (Ellis et al. 2007; Krahe et al. 2008; Mehaffey et al. 2008b).

Natural electrosensory stimuli comprise those caused by prey which are typically localized within a small region of the animal's skin (Nelson and MacIver 1999) as well as those caused by conspecifics which are typically diffuse and impinge on most if not all of the skin surface (Chacron et al. 2011). Previous studies have found strong evidence for parallel processing in ELL as pyramidal cells from different maps are tuned to different temporal frequency components of electrosensory stimuli (Shumway 1989a; Krahe et al. 2008) (see Chacron et al. 2011; Krahe and Maler 2014 for review). Indeed, pyramidal cells within LS respond best to high‐frequency (>40 Hz) electrocommunication stimuli (Krahe et al. 2008; Marsat et al. 2009; Marsat and Maler 2010) (see (Marsat et al. 2012) for review) whereas CMS cells respond best to low‐ frequency (<10 Hz) stimuli caused by same‐sex conspecifics (Shumway 1989a; Krahe et al. 2008). Thus, while these results argue for a functional organization across maps in that they each mediate behavioral responses to different classes of electrosensory stimuli (Metzner and Juranek 1997; Maler 2009a,b), it is important to realize that these were obtained using stationary stimuli.

Motion is a key component of the natural electrosensory environment that gives rise to important behavioral responses (Nelson and MacIver 1999; Cowan and Fortune 2007; Metzen and Chacron 2014). Given that ELL pyramidal neurons respond to moving objects (Bastian 1981; Chacron et al. 2009), we tested whether the functional organization of ELL maps uncovered for stationary stimuli would apply to motion stimuli as well. We used moving objects with a wide range of behaviorally relevant velocities from 2.5 to 30 cm/sec. These roughly correspond to temporal frequencies between 2.5 and 30 Hz, which is within the range where important differences in frequency tuning were previously observed using stationary stimuli (Krahe et al. 2008).

## Methods

### Animals

For this study, we used the weakly electric fish *Apteronotus leptorhynchus*. Animals were obtained from tropical fish suppliers and were housed in laboratory tanks. The fish were well acclimated to their new environment during several days before their participation in experiments. Animal husbandry was done according to published guidelines (Hitschfeld et al. 2009). McGill University's animal care committee approved all husbandry and surgical procedures.

### Surgery

The experimental procedures were similar to those used previously (Krahe et al. 2008; Toporikova and Chacron 2009; Deemyad et al. 2013). The animals were first immobilized by intramuscular injection of tubocurarine (0.2 mg; Sigma–Aldrich, St. Louis, MO) before being moved to the experimental tank where they were respirated with oxygenated water via a mouth tube at a flow rate of ~10 mL/min. After local anesthesia with 2% lidocaine, ~6 mm^2^ of skin was removed in order to expose the skull and a metal post was glued with cyanoacrylate to the exposed area for stabilization. We then drilled a hole of ~2 mm^2^ in the skull above the hindbrain. The surface of the brain was covered by saline throughout the experiment.

### Recordings and stimulation

Recordings from ELL neurons were made by using metal‐filled micropipettes with a tip diameter of ~5 micrometer which were plated with gold and platinum (Frank and Becker 1964). We used surface landmarks as well as the recording depth in order to assign a given recording as belonging to either of CMS, CLS, and LS as previous studies have found such to be adequate (Krahe et al. 2008). The stimulus consisted of a 2 cm wide metal bar back coated with plastic that was moved by a pen plotter. This object moved back and forth along the animal's rostro‐caudal axis over a distance of 20 cm roughly 1 cm away from the animal as done previously (Chacron et al. 2009; Chacron and Fortune 2010; Khosravi‐Hashemi et al. 2011; Vonderschen and Chacron 2011; Khosravi‐Hashemi and Chacron 2012). The stimulation protocol consisted of six movement stimuli each lasting 200 sec with average rms velocities of 2.5 cm/sec, 5 cm/sec, 10 cm/sec, 15 cm/sec, 20 cm/sec, and 30 cm/sec. These values were chosen to span the behaviorally relevant range observed during prey capture and refuge tracking behaviors (Bastian 1982; Rose and Canfield 1993a,b; Nelson and MacIver 1999; Cowan and Fortune 2007). Each stimulus velocity roughly corresponds to temporal frequencies of 2.5 Hz, 5 Hz, 10 Hz, 15 Hz, 20 Hz, and 30 Hz in increasing order assuming a Gaussian electric image of 1 cm standard deviation (Bastian et al. 2002; Chen et al. 2005).

Previous studies have identified two classes of ELL pyramidal cells: basilar and nonbasilar (Maler 1979; Maler et al. 1981), which also are referred to as E and I cells because they respond to increased EOD amplitude with excitation and inhibition, respectively (Saunders and Bastian 1984). Thus, a given pyramidal cell can be reliably identified as belonging to either class through their responses to sensory input. Here, we used a Gaussian white noise stimulus (120 Hz cutoff, 8th order Butterworth) that was applied as a modulation of the fish's own EOD globally via chloridized silver wire electrodes positioned 15 cm away from the fish on either side of the animal as described previously (Chacron et al. 2003; McGillivray et al. 2012). We then computed the reverse correlation between the stimulus and spike train (i.e., the spike triggered average), and determined whether the neuron responded to increases or decreases in the stimulus as done previously (Chacron et al. 2005b; Chacron 2006; Avila Akerberg et al. 2010; Avila Akerberg and Chacron 2011).

### Analysis

Data were acquired with Cambridge Electronic Design Power1401 hardware and Spike2 software (Cambridge, UK) and were analyzed using Spike2 (CED) and custom‐made routines in MATLAB (The Mathworks, Natick, MA). The action potential times were obtained by thresholding the high‐pass filtered (400‐Hz cutoff) recorded signal. The spike train obtained from signal recorded under moving object stimulation was used to generate peristimulus time histograms (PSTHs) in response to each velocity separately. For each velocity, the full cycle of movement was divided into 40 bins, this corresponds to binwidths of 500 ms, 250 ms, 125 ms, 71.43 ms, 62.5 ms, 41.67 ms, for 2.5 cm/sec, 5 cm/sec, 10 cm/sec, 15 cm/sec, 20 cm/sec, 30 cm/sec, respectively. To determine whether the obtained responses were actually due to the moving object stimulus rather than random firing rate fluctuations, we obtained surrogate datasets by simulating a Poisson process with the same mean firing rate as the cell for the same length of time. This was done 10,000 times in order to obtain a probability distribution of firing rate response. We empirically found that this distribution was well fitted by a Gaussian and generated a confidence interval by taking three times the standard deviation of the distribution. Therefore, firing rate responses either above or below the confidence interval were deemed extremely unlikely (*P* < 0.0002) to be caused by random fluctuations and thus assumed to be caused by the moving object stimulus. In practice, only the large increases/decreases in firing rate were caused by the moving object. Thus, we calculated the response for each velocity as the difference between the maximum and minimum values of the PSTH divided by its mean value. In some figures, the responses were normalized to their maximum value across velocities in order to better illustrate key features.

In order to determine whether ELL pyramidal cells displayed directionally selective responses, we computed a directional selectivity index (DSI) given by the following equation (Chacron et al. 2009; Chacron and Fortune 2010; Khosravi‐Hashemi et al. 2011; Vonderschen and Chacron 2011; Khosravi‐Hashemi and Chacron 2012):

$$DSI=\frac{|R_{rc}-R_{cr}|}{\max(R_{rc},R_{cr})}$$

where *R*_*rc*_ is the response when the object moves from rostral to caudal and *R*_*cr*_ is the response when the object moves from caudal to rostral. Thus, we have DSI = 0 for a neuron that responds equally well to movement in either direction and DSI = 1 in the case of a neuron that only responds to movement in one direction. However, fluctuations and estimation errors will erroneously give rise to positive values of DSI even when simulating processes that are nondirectionally selective by definition (Chacron et al. 2009). Thus, in order to test whether the DSI values obtained for our dataset were significant, we tested whether the responses when the object moves from rostral to caudal as compared to when the object moves from caudal to rostral were significantly different from one another by computing their difference and comparing it to twice the confidence interval.

### Model

Since the experiments have been done in 1D (rostro‐caudal axis), the moving stimulus and the receptive field were also modeled in 1D. Specifically, the moving stimulus was modeled as a moving Gaussian image consistent with previous studies (Chen et al. 2005; Maler 2009a):

$$S\left(x,t\right)=Aexp\left(-\frac{{\left(x-vt\right)}^{2}}{2\theta^{2}}\right)$$

Where A is the peak amplitude, *θ* is the standard deviation, and v is the velocity of the moving object.

We modeled the receptive fields of ELL pyramidal cells based on experimental studies showing antagonistic center–surround organization (Bastian et al. 2002) using Gabor filters:

$$RF\left(x\right)=\frac{1}{\sqrt{2\pi }\sigma_{E}}*\exp\left(-\frac{{\left(x-x_{max}/2\right)}^{2}}{{2\sigma_{E}}^{2}}\right)-\frac{1}{\sqrt{2\pi }\sigma_{1}}*\exp\left(-\frac{{\left(x-x_{max}/2\right)}^{2}}{{2\sigma_{1}}^{2}}\right)$$

Where *σ*_E_ and *σ*_1_ define the size of the center and surround zones, respectively. The center, *F*_*c*_(*t*), and surround, *F*_*s*_(*t*), contributions to the cell's firing rate in response to the moving object were computed by integrating across position *x* for a given time *t*:

$$F_{c}\left(t\right)=\int_{x_{c}}^{}S\left(x,t\right)RF\left(x\right)dx$$

$$F_{s}\left(t\right)=\int_{x_{s}}^{}S\left(x,t\right)RF\left(x\right)dx$$

$$Fr\left(t\right)=F_{c}\left(t\right)+F_{s}\left(t-d\right)+R_{b}$$

Where *x*_*c*_ is the range of values for *x* in which the *RF*(*x*) is positive while *x*_*s*_ is the range for which *RF*(*x*) is negative. The cell's time varying firing rate is then given by:

$$fr\left(t\right)=F_{c}\left(t\right)+F_{s}\left(t-d\right)+R_{b}$$

Where the *fr*(*t*) is the time‐varying firing rate of the ELL model neuron, *d* is the time delay between the responses of the center and surround zones in order to account for axonal transmission delays, and *R*_*b*_ is the baseline (i.e., in the absence of stimulation) firing rate which was set to 15 Hz. We analyzed the model's output in the same way as described above for the experimental data. Parameter values used for the different cell types are given below with A = 75, *R*_*b*_ = 15 Hz:

**Table:** 

	Center size (cm)	Surround Size (cm)	Delay (ms)
CMS E‐cell	0.07	0.6	30
CMS I‐cell	0.09	0.6	30
CLS E‐cell	0.15	0.6	20
CLS I‐cell	0.1	0.5	20
LS E‐cell	0.25	0.27	15
LS I‐cell	0.2	0.25	15

## Results

### ELL Pyramidal cells display velocity sensitivity

In order to investigate how both E‐ and I‐type (Fig. 1B) ELL pyramidal cells across the three tuberous segments (Fig. 1A) respond to moving objects, we performed extracellular recordings while moving an object back and forth along the body of the fish with various velocities (Fig. 1C, D). Our dataset consists of *N* = 19 CMS (10 E‐type and 9 I‐type), *N* = 25 CLS (11 E‐type and 14 I‐type), and *N* = 17 LS (10 E‐type and 7 I‐type) pyramidal cells.

The responses of typical CLS I‐ and E‐type pyramidal cells to moving objects are shown in Fig. 1C, D, respectively. Pyramidal cells tended to fire their baseline (i.e., in the absence of stimulation) firing rates except when the moving object is at a particular position on the animal's body where the I‐cell responds with inhibition (Fig. 1C) and the E‐cell responds with excitation (Fig. 1D). These responses were similar across repeated trials as shown by raster plots (Fig. 1E, F). We thus averaged across trials to obtain the neurons' time‐dependent firing rate (i.e., peristimulus time histogram or PSTH) (Fig. 1G, H). We used surrogate datasets in order to determine whether the observed features in the PSTH were due to the moving object or simply due to random firing rate fluctuations by generating a confidence interval around the cell's baseline firing rate (gray bands in Fig. 1G, H). Firing rates outside the confidence interval were extremely unlikely (*P* < 0.002) to be caused by random firing rate fluctuations and were thus assumed to instead be caused by the moving object stimulus. We empirically found that these corresponded to the negative/positive deflections in firing rate for I and E cells, respectively (Fig. 1G, H).

**Figure 2:**
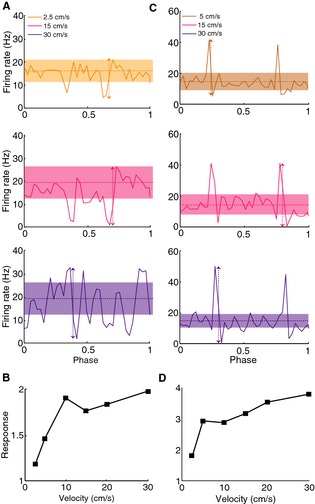
ELL pyramidal cells respond differentially to an object moving at different velocities. (A) PSTH responses from an example CLS I‐cell to an object moving with velocities 2.5 cm/sec (top), 15 cm/sec (middle), and 30 cm/sec (bottom). The vertical arrows show the difference between the maximum and minimum firing rates while the horizontal lines show the mean firing rate. The bands show the confidence intervals generated from surrogate datasets as described in the methods. Firing rates outside the confidence interval were taken as caused by the stimulus. (B) Velocity tuning curve from this same I‐cell showing increasing response as a function of increasing velocity. (C) PSTH responses from an example CLS E‐cell to an object moving with velocities 5 cm/sec (top), 15 cm/sec (middle), and 30 cm/sec (bottom). The vertical arrows show the difference between the maximum and minimum firing rates while the horizontal lines show the mean firing rate. The bands show the confidence intervals generated from surrogate datasets as described in the methods. Firing rates outside the confidence interval were taken as caused by the stimulus and mostly corresponded to the peaks or troughs. (D) Velocity tuning curve from this same E‐cell showing increasing response as a function of increasing velocity.

We next varied the average velocity of the moving object. We observed that the firing rate modulation due to object movement generally increased with object velocity for these same I‐type (Fig. 2A) and E‐type (Fig. 2B) pyramidal cells. As only these modulations were outside the confidence intervals, we quantified the cell's response to the moving object by taking the difference between the maximum and minimum values of the firing rate and dividing the result by the mean firing rate response. Thus, when considering this measure, both E‐ and I‐type pyramidal neurons display high‐pass velocity sensitivity profiles as shown in Fig. 2B, D, respectively. We also observed that the phase at which the peak/through response occurred at increased as a function of velocity (Fig. 2A, C), indicating that there is a greater response lag for larger velocities.

### ELL pyramidal cells across segments display similar velocity sensitivity

As mentioned above, previous studies have shown that pyramidal cells from these three maps displayed significant differences in their frequency tuning to either stationary sensory stimulation (Shumway 1989a; Krahe et al. 2008) and intracellular current injection (Mehaffey et al. 2008b) as recently reviewed (Krahe and Maler 2014). Based on the fact that velocity is distance over time and that time is the inverse of frequency, one might expect that the differences in frequency tuning observed using stationary stimuli would translate to responses to moving objects as velocity would then be proportional to frequency. We used velocities between 2.5 cm/sec and 30 cm/sec, which roughly corresponds to temporal frequencies between 2.5 Hz and 30 Hz assuming a Gaussian electric image with 1 cm standard deviation and previous studies (Krahe et al. 2008) have demonstrated significant differences between the tuning of CMS, CLS, and LS pyramidal cells over this frequency range.

**Figure 3:**
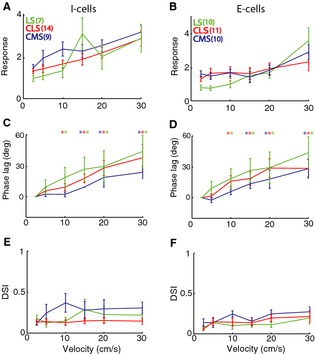
ELL pyramidal cells across CMS, CLS, and LS display similar velocity tuning curves. (A) Population‐averaged velocity tuning curves for I cells from LS (green), CLS (red), and CMS (blue). Note that the curves did not differ significantly from one another (*P* > 0.05, one‐way ANOVA) at all velocities. (B) Population‐averaged velocity tuning curves for E cells from LS (green), CLS (red), and CMS (blue). Note that the curves did not differ significantly from one another (*P* > 0.05, one‐way ANOVA) at all velocities except at 2.5 cm/sec in which CMS cells displayed stronger response compared to LS cells (*P* = 0.03, one‐way ANOVA). Error bars show 1 SEM. (C) Population‐averaged phase lag difference (relative to 2.5 cm/sec) as a function of velocity for I cells across segments. Note that the curves did not differ significantly from one another (*P* > 0.05, one‐way ANOVA) at all velocities. (D) Population‐averaged phase lag difference (relative to 2.5 cm/sec) as a function of velocity for E cells across segments. Note that the curves did not differ significantly from one another (*P* > 0.05, one‐way ANOVA) at all velocities. For C,D, we observed larger phase lags for larger velocities as the phase lag difference were significantly different from zero (“*”) as the *P* = 0.05 level using a sign rank test. (E) Population‐averaged directional selectivity index (DSI) values as a function of velocity for I cells across segments. Note that the curves did not differ significantly from one another (*P* > 0.05, one‐way ANOVA) at all velocities. (F) Population‐averaged DSI values as a function of velocity for E cells across segments. Note that the curves did not differ significantly from one another (*P* > 0.05, one‐way ANOVA) at all velocities. For E,F, all values of DSI were not significantly different from zero (*P* > 0.05, see methods).

In order to test this prediction, we compared the responses of the pyramidal neurons within CMS, CLS, and LS to moving objects with different velocities. Figure 3A, B show the population‐averaged response functions for I‐ and E‐type pyramidal cells for CMS (blue), CLS (red), and LS (green) segments, respectively. Overall, we found that cell types across segments displayed similar tuning curves that were not significantly different from one another (*P* > 0.01, one‐way ANOVAs). In general, responses increased as a function of velocity. Furthermore, the phase lag also increased as a function of velocity for all cell types across segments (Fig. 3C, D) and was not significantly different across segments (*P* > 0.01 one‐way ANOVAs). These results demonstrate that despite showing important differences in frequency tuning to stationary stimuli, electrosensory pyramidal neurons across segments and classes displayed remarkably similar tuning to moving objects with varying velocities.

Previous studies have shown that ELL pyramidal cells respond to moving objects in a directionally unselective manner (Bastian 1981; Chacron et al. 2009). We thus tested whether this was the case for our dataset by computing the directional selectivity index (DSI) that is equal to zero for a nondirectionally selective neuron and one in the case of a neuron that responds only to movement in one direction. We found low values of DSI across our dataset (DSI = 0.17 ± 0.01), consistent with previous studies (Chacron et al. 2009), and the maximum response in one direction was not significantly different than that obtained for the other direction for all cells in our dataset and for all stimuli. The population‐averaged DSI values are summarized in (Fig. 3E, F) and were not significantly different across segments for all velocities (*P* > 0.01, one‐way ANOVAs).

### Center–surround receptive field structure accompanied by delay in surrounds response can qualitatively reproduce the observed high‐pass velocity tuning profiles

**Figure 4:**
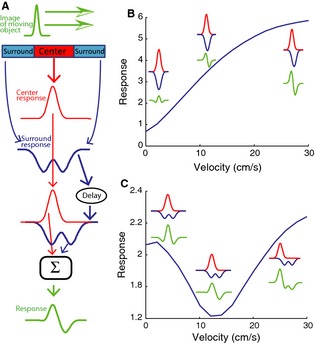
Modeling ELL pyramidal cell responses to moving objects. (A) Model schematic in which the receptive field's antagonistic center–surround organization is modeled in one dimension using Gabor filters. The electric image caused by the object was modeled as a moving Gaussian (green). The contributions of the center (red) and surround (blue) are obtained by convolving the moving stimulus with the appropriate Gabor filters. The surround response is then delayed and the two contributions are summed and a constant baseline added in order to obtain the total response to the moving object. (B) Velocity tuning curve obtained from the model when the width of the moving object stimulus is bigger than that of the receptive field center. The responses of the center and surround are shown in red and blue, respectively. The total response is shown in green. Parameter values used were *θ* = 0.2 cm, A = 75, *σ*_E_ = 0.02 cm, *σ*_I_ = 0.2 cm, del = 20 ms. (C) Velocity tuning curve obtained from the model when the width of the moving object stimulus is similar to that of the receptive field center. The responses of the center and surround are shown in red and blue, respectively. The total response is shown in green. Parameter values used were *θ* = 0.1 cm, A = 75, *σ*_E_ = 0.09 cm, *σ*_I_ = 0.2 cm, del = 20 ms.

In order to test whether the differential receptive field organization of electrosensory pyramidal neurons across segments could explain the observed velocity response profiles, we built a simple mathematical model of ELL pyramidal cells (Fig. 4A). The model consists of an antagonistic center–surround receptive field with the surround response delayed with respect to that of the center in order to account for the fact that previous studies have shown that delayed feedback contributes to the known receptive field properties of these neurons (Chacron et al. 2003, 2005b; Chacron 2006). We found that, depending on parameter values, our model can give rise to qualitatively different velocity tuning profiles (Fig. 4B, C).

For clarity, we only consider the case of an E‐type cell below as the arguments are exactly the same for an I‐type cell if excitation is replaced by inhibition and vice versa. For smaller receptive field sizes compared to the size of the electric image caused by the moving object, the responses from both parts of the surround coalesce into a bell‐shaped inhibition. The relative delay between the center excitation and the surround inhibition increases with velocity, thereby allowing the net excitation followed by inhibition to get progressively larger, thereby causing larger changes in firing rate and therefore increasing the response measure (Fig. 4B). In contrast, when the receptive field size is large relative to the object size, the response of the surround zone is instead bimodal in shape (Fig. 4C). For low object velocities, the delay between the center and surround responses is negligible and the trough of the surround response is temporally aligned with the peak of the center, thereby causing greater excitation. For larger velocities, the delay between center and surround becomes larger. In particular, for an intermediate value of delay, the first peak of the surround response is temporally aligned with the center response, thereby weakening the net excitation and causing a decreased response. For yet higher velocities, the delay increases and the net excitation increases as it is not partially occluded by inhibition anymore and therefore the response increases (Fig. 4C).

**Figure 5:**
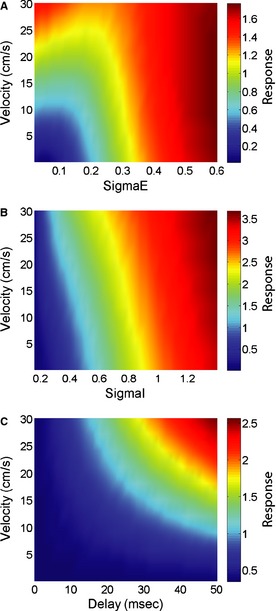
Velocity tuning curves strongly depend on receptive field properties as predicted by our model. (A) Response as a function of velocity and center width while keeping the surround width constant. Other parameter values used were *θ* = 0.75 cm, A = 75, *σ*_I_ = 1 cm, del = 20 ms. (B) Response as a function of velocity and surround width while keeping the center width constant. Other parameter values used were *θ* = 0.75 cm, A = 75, *σ*_E_ = 0.15 cm, del = 20 ms. (C) Response as a function of velocity and delay. Other parameter values used were *θ* = 0.75 cm, A = 75, *σ*_E_ = 0.15 cm, *σ*_I_ = 0.3 cm.

We next systematically varied the model parameters and computed the response to the moving object. First, we varied the size of the center zone of the receptive field while keeping the surround size constant at 1 cm and the delay constant at 20 ms as well as the object's velocity (Fig. 5A). Overall, stronger responses were observed for higher velocities as explained above. Moreover, the highest responses observed for small receptive field size, thereby allowing a greater surround response (Fig. 5A). We next varied the surround size while keeping the center zone size constant at 0.15 cm and the delay fixed at 20 ms. Again, higher responses were observed for higher velocities relative to those observed for lower velocities for a given surround size (Fig. 5B). Moreover, stronger responses were observed for increasing surround size, thereby allowing a greater surround response. Thus, a greater mismatch between the relative strength of center and surround will in general give rise to a greater response to a moving object. Finally, we varied the delay between the surround and center responses while keeping the center and surround sizes fixed at 0.15 cm and 0.3 cm, respectively (Fig. 5C). We again observed higher responses to higher velocities for a given nonzero delay. Moreover, responses to moving objects became greater overall for increasing delays, thereby causing a high‐pass velocity tuning profile. These results show that our model can qualitatively reproduce the high‐pass velocity tuning profiles observed experimentally.

### Known anatomical and physiological differences in pyramidal neuron receptive field organization can explain the differences in the velocity tuning profiles observed experimentally

We next tested whether the known differences in receptive field organization across the segments and between E‐ and I‐type pyramidal cells could explain differences between their velocity response tuning profiles to moving objects as observed experimentally. To do so, we selected parameter values that were consistent with the following experimental observations: (1) the receptive field center size of CMS cells is smaller than that of CLS cells, which is smaller than that of LS cells (Shumway 1989a; Maler 2009a); (2) I cells have a smaller receptive field center size than E cells (Bastian et al. 2002); (3) the feedback delay for CMS neurons is larger than that for CLS neurons, which is larger than that for LS neurons (Maler 1979).

**Figure 6:**
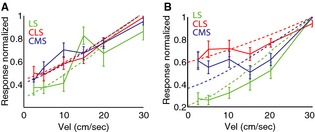
A simple model of ELL pyramidal cell receptive field organization can reproduce experimental data. (A) Population‐averaged normalized velocity tuning curves for LS (solid green), CLS (solid red), and CMS (solid blue) I cells with corresponding model fits (dashed lines). (B) Population‐averaged normalized velocity tuning curves for LS (solid green), CLS (solid red), and CMS (solid blue) E cells with corresponding model fits (dashed lines). Error bars show 1 SEM.

In order to better emphasize the differences across segments for a given cell type, the experimentally observed velocity tuning profiles were normalized to their maximum value (Figs 6A, B). The response profiles of I cells across segments remained quite similar after normalization (Fig. 6A). In contrast, normalization helps to emphasize that the velocity tuning profile of LS E cells increases at a faster rate as a function of velocity than that of CMS E cells, which itself increases at a faster rate than that of CLS E cells (Fig. 4B). Our results show that using parameter values consistent with known anatomical and physiological differences between the ELL segments, our model can quantitatively reproduce the differences in velocity tuning observed experimentally. Thus, we conclude that the differences between the observed velocity tuning response profiles of pyramidal neurons across segments can be largely if not exclusively explained by differences in their receptive field organization.

### ELL pyramidal neurons display high‐pass velocity tuning to moving objects with different widths as predicted by our model

**Figure 7:**
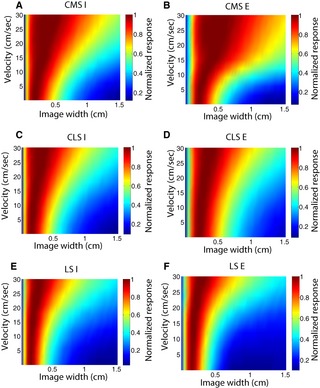
Velocity tuning curves of ELL pyramidal neurons are predicted to not qualitatively depend on object width for a wide range of values. Response as a function of velocity and image width for CMS I cells (A), CMS E cells (B), CLS I cells (C), CLS E cells (D), LS I cells (E), and LS E cells (F).

In order to gain understanding as to how ELL pyramidal neurons respond to moving objects with different sizes, we varied the width of the moving object in our model while keeping all other parameters constant. We found that model E‐ and I‐type pyramidal neurons across the CMS, CLS, and LS segments displayed high‐pass velocity tuning profiles to a significant range of object widths (Fig. 7).

## Discussion

### Summary of results

We investigated ELL pyramidal neuron responses to moving objects with varying velocity across parallel maps of the body surface. Given the known and important differences seen across maps in terms of responses to stationary stimuli and receptive field structure, we expected to find major differences in their responses to moving objects. However, to our surprise, we found that all pyramidal cells, regardless of type of map, responded most strongly to objects moving at larger velocities as quantified by changes in firing rate. To gain intuition as to this surprising result, we built a mathematical model incorporating the known antagonistic center–surround organization of ELL pyramidal neurons. We found that when the surround response was delayed with respect to that of the center in order to account for finite axonal conduction velocities, our model could successfully reproduce the high‐pass velocity tuning curves observed experimentally. Moreover, we found that when the known differences in receptive field structure are taken into account, our model could quantitatively reproduce the experimentally observed differences across cell types and maps. Our model further predicted high‐pass velocity tuning curves for objects of different sizes. We conclude that contrary to what was seen previously for stationary stimuli, weakly electric fish do not appear to take advantage of parallel pathways in order to process specific features of motion stimuli.

### Pyramidal cell receptive field organization rather than intrinsic properties primarily determines velocity tuning to lateral motion

Our modeling results predict that feedback input onto ELL pyramidal cells strongly contributes to their receptive field organization. ELL pyramidal cells receive multiple sources of feedback (Berman and Maler 1999). While previous work has shown that the indirect feedback input onto pyramidal cells attenuates their response to low‐frequency stimulation and is only elicited by spatially diffuse stimuli (Bastian 1986, 1999; Chacron et al. 2003, 2005b; Bastian et al. 2004; Chacron 2006; Bol et al. 2011, 2013), the functional role of the direct feedback input has not been elucidated yet (but see Berman and Maler 1999). Previous studies have furthermore shown that the indirect feedback contributes to shaping the receptive field properties of ELL pyramidal cells by extending the surround (Chacron et al. 2003, 2005a), which was incorporated in the modeling component of this study. Thus, our results point to a new function for indirect feedback input onto ELL pyramidal cells: to improve responses to higher velocities relative to those at lower velocities. Further studies using pharmacological blockade of feedback pathways are needed to verify these predictions, however. In fact, our results show that the velocity tuning curves of ELL pyramidal cells critically depend on receptive field structure, which is similar to that proposed for velocity‐sensitive neurons in the visual system (Rodieck 1965).

This finding is surprising and is in stark contrast to results obtained using stationary stimuli showing large differences in ELL pyramidal cell responses across different maps (Krahe et al. 2008; Marsat et al. 2009, 2012; Marsat and Maler 2010; Chacron et al. 2011; Krahe and Maler 2014) that appear to be mostly due to differences in the presence of specific ion channels (Ellis et al. 2007, 2008; Mehaffey et al. 2008a,b) rather than differences in receptive field size (Shumway 1989b; Maler 2009a). Thus, results obtained using stationary stimuli cannot be used in general to infer response to motion stimuli because the former will simultaneously while the latter will instead sequentially stimulate the receptive field. This said, the minor differences seen across E cells might be due to differential adaptation. Indeed, previous studies have shown that LS E cells displayed faster adaptation than CMS E cells (Krahe et al. 2008). As adaptation can lead to high‐pass filtering (Benda and Herz 2003; Benda and Hennig 2008; Deemyad et al. 2012), it is thus likely that the steeper velocity tuning curves seen in LS E‐type pyramidal cells would be in part due to faster adaptation. Recent studies have shown that SK channels lead to adaptation in ELL pyramidal neurons (Ellis et al. 2007) and that these are most present in LS E cells (Ellis et al. 2008). It is thus likely that the greater density of SK channels found in LS E cells contribute to making their velocity tuning curves steeper than those of CMS cells.

A recent study has demonstrated that adaptation in electroreceptor neurons led to velocity invariant tuning curves for looming motion (Clarke et al. 2013). While our results show that such velocity invariance is not seen for their postsynaptic targets, ELL pyramidal neurons, it is important to realize that our study used lateral rather than looming motion. Further studies are needed to characterize and explain ELL pyramidal cell responses to looming motion. While compensatory mechanisms exist in the retina to lead to velocity invariant tuning curves (Trenholm et al. 2013), no evidence for such mechanisms was found in ELL pyramidal cells as the phase lag increased with velocity (Figs 2, 3). It is nevertheless possible that such mechanisms might be found in higher brain areas.

### Consequences for behavior

Behavioral studies have shown that motion is an important component of the natural electrosensory environment. First, weakly electric fish can detect and then track the location of a small prey during capture behavior (Nelson and MacIver 1999). Second, weakly electric fish can track the back and forth movement of a refuge in order to stay inside (Heiligenberg 1973; Bastian 1982; Rose and Canfield 1993a,b; Cowan and Fortune 2007). Third, a recent behavioral study has shown that information as to the detailed time course of the second order features of natural electrosensory stimuli that occur during movement is retained in the brain (Metzen and Chacron 2014). These behaviors all require that fish track the detailed time course of changes in electrosensory stimuli that occur during movement. In particular, kinematic analysis has shown that refuge tracking is best for low movement velocities but that neurons that are best tuned to higher velocities are needed to control this behavior because of inertia (Cowan and Fortune 2007). Our results confirm these predictions by showing that ELL pyramidal neurons are indeed best tuned to higher velocities.

In contrast, prey capture behavior studies have shown greatest success for lower velocities around 10 cm/sec (Nelson and MacIver 1999). Our object consisted of a moving metal bar with relatively large width (2 cm) and thus did not accurately mimic electric images caused by prey. However, our modeling predicts that the high‐pass velocity tuning observed experimentally will be seen for electrical images with different spatial extents. We further note that qualitatively similar results were obtained when we used a moving bar with smaller width (0.1 cm, data not shown). Thus, it is likely that results similar to our own will be obtained when using moving objects whose electric image matches that caused by prey. Further studies are needed, however, to verify this hypothesis.

### Velocity tuning in higher brain areas

ELL pyramidal neurons project to the midbrain Torus semicircularis (TS). Previous studies have shown that TS neurons tend to respond more selectively to stimuli than ELL neurons (Vonderschen and Chacron 2009, 2011; McGillivray et al. 2012). In particular, directional selectivity emerges at the level of TS (Chacron et al. 2009; Chacron and Fortune 2010). While the velocity tuning curves of TS neurons have not been investigated to date, our results show that pyramidal cell heterogeneities across maps are unlikely to contribute to putative heterogeneities in TS neurons. Further studies should investigate velocity tuning in TS.

## Conclusion

We investigated whether parallel coding was used as a strategy to encode lateral movement in ELL pyramidal neurons. We found only minor differences across cell types and across parallel maps of the body surface and all cells displayed preference for higher velocities. These can be attributed to the receptive field structure of ELL pyramidal neurons and to delayed feedback. Thus, our results show that parallel coding is most likely not used for lateral motion, unlike stationary stimuli.

## Conflict of Interest

None declared.
